# Human Papillomavirus Genotype Prevalence in Invasive Penile Cancers from a Registry-Based United States Population

**DOI:** 10.3389/fonc.2014.00009

**Published:** 2014-02-05

**Authors:** Brenda Y. Hernandez, Marc T. Goodman, Elizabeth R. Unger, Martin Steinau, Amy Powers, Charles F. Lynch, Wendy Cozen, Maria Sibug Saber, Edward S. Peters, Edward J. Wilkinson, Glenn Copeland, Claudia Hopenhayn, Youjie Huang, Meg Watson, Sean F. Altekruse, Christopher Lyu, Mona Saraiya, The HPV Typing of Cancer Workgroup

**Affiliations:** ^1^University of Hawaii Cancer Center, University of Hawaii, Honolulu, HI, USA; ^2^Division of High-Consequence Pathogens and Pathology, National Center for Emerging and Zoonotic Infectious Diseases, Centers for Disease Control and Prevention, Atlanta, GA, USA; ^3^Department of Epidemiology, College of Public Health, The University of Iowa, Iowa City, IA, USA; ^4^Departments of Preventive Medicine and Pathology, USC Keck School of Medicine, Norris Comprehensive Cancer Center, University of Southern California, Los Angeles, CA, USA; ^5^Department of Epidemiology, School of Public Health, Louisiana State University Health Sciences Center, New Orleans, LA, USA; ^6^Department of Pathology, Immunology, and Laboratory Medicine, College of Medicine, University of Florida, Gainesville, FL, USA; ^7^Michigan Department of Community Health, Lansing, MI, USA; ^8^Department of Epidemiology, College of Public Health, University of Kentucky, Lexington, KY, USA; ^9^Florida Department of Health, Tallahassee, FL, USA; ^10^Division of Cancer Prevention and Control, National Center for Chronic Disease Prevention, and Health Promotion, Centers for Disease Control and Prevention, Atlanta, GA, USA; ^11^Division of Cancer Control and Population Sciences, National Cancer Institute, Rockville, MD, USA; ^12^Battelle Memorial Institute, Durham, NC, USA

**Keywords:** human papillomavirus, HPV, prevalence, penile cancer, United States

## Abstract

**Background:** Human papillomavirus (HPV) is estimated to play an etiologic role in 40–50% of penile cancers worldwide. Estimates of HPV prevalence in U.S. penile cancer cases are limited.

**Methods:** HPV DNA was evaluated in tumor tissue from 79 invasive penile cancer patients diagnosed in 1998–2005 within the catchment areas of seven U.S. cancer registries. HPV was genotyped using PCR-based Linear Array and INNO-LiPA assays and compared by demographic, clinical, and pathologic characteristics and survival. Histological classification was also obtained by independent pathology review.

**Result**s: HPV DNA was present in 50 of 79 (63%) of invasive penile cancer cases. Sixteen viral genotypes were detected. HPV 16, found in 46% (36/79) of all cases (72% of HPV-positive cases) was the most prevalent genotype followed equally by HPV 18, 33, and 45, each of which comprised 5% of all cases. Multiple genotypes were detected in 18% of viral positive cases. HPV prevalence did not significantly vary by age, race/ethnicity, population size of geographic region, cancer stage, histology, grade, penile subsite, or prior cancer history. Penile cases diagnosed in more recent years were more likely to be HPV-positive. Overall survival did not significantly vary by HPV status.

**Conclusion:** The relatively high prevalence of HPV in our study population provides limited evidence of a more prominent and, possibly, increasing role of infection in penile carcinogenesis in the U.S. compared to other parts of the world.

## Introduction

The annual, average age-adjusted incidence of invasive penile cancer in the U.S. is less than 1 per 100,000, which represents less than 0.5% of all cancers in men (1). Penile cancer incidence varies globally, with lower incidence in the United States and other Western countries and comparably higher incidence in parts of Africa, Southeast Asia, and Latin America (2, 3). Human papillomavirus (HPV) likely represents a distinct etiologic pathway of penile carcinogenesis (4). Worldwide, HPV is estimated to play an etiologic role in 40–50% of penile cancers (2, 3, 5). HPV 16 is the most frequently detected genotype in penile tumors (2, 6, 7). Other risk factors for penile cancer include cigarette smoking, lack of circumcision, poor hygiene, and phimosis (4, 8–11). The extent to which worldwide differences in penile cancer incidence are attributed to variation in HPV prevalence is unknown. There is some evidence of regional differences in HPV prevalence in penile tumors (2). To date, however, there are limited estimates of the prevalence of HPV in U.S. penile cancer patients. In U.S. studies, including small case series from single institutions and single geographic regions, prevalence ranged from 31 to 82% (12–15).

## Materials and Methods

We conducted a study to evaluate the genotype-specific HPV distribution in invasive penile cancer cases from the U.S. and to compare the viral status of cases by demographic, clinical, and pathologic characteristics. This evaluation was part of a larger initiative by the Centers for Disease Control and Prevention (CDC) to examine HPV genotype distribution among anogenital and select head and neck cancer sites for cases diagnosed in the United States prior to the implementation of widespread HPV prophylactic vaccination (16). This study was approved by the institutional review boards overseeing the CDC and participating cancer registries. Invasive penile cancer cases were defined based on the International Classification of Diseases for Oncology Version 3 (17). Cases were selected from patients with histologically confirmed invasive penile cancer who were diagnosed in Kentucky, Louisiana, Michigan, Iowa, Hawaii, and Los Angeles County. Additionally, Florida included a three-county catchment area. Penile cancer cases from Iowa, Hawaii, and Los Angeles County were selected from those with archival tissue in existing tissue repositories. The three residual tissue repositories (RTR) (18) are a part of the National Cancer Institute’s (NCI) surveillance, epidemiology, and end-results (SEER) program and consist of collections of deidentified formalin-fixed paraffin-embedded (FFPE) tumor tissue specimens obtained from area pathology laboratories. Penile cancer cases from Florida, Kentucky, Louisiana, and Michigan were sampled from all invasive cases diagnosed in 2004–2005 with available FFPE tissue from area pathology laboratories. Penile cases from Hawaii included all RTR cases diagnosed in 2000–2004; Iowa and Los Angeles County cases included those diagnosed in 1998–2000. A total of 163 penile cancer cases were initially identified across the seven registries and tumor tissue specimens were available for 90.

Formalin-fixed paraffin-embedded tissue specimens from the 90 cases were prepared at central laboratories servicing each registry or medical facility following a uniform protocol. As previously detailed, one representative block from each case was prepared using procedures to minimize the risk of sample-to-sample contamination (16). Sections were prepared from each block using a new disposable blade for each case. The first and last sections were stained with hematoxylin and eosin (H&E) and intervening sections were transferred into 2 ml conical tubes (Simport, Beloeil, Canada). Prepared FFPE tissues were sent to the CDC. H&E sections were reviewed by a study pathologist (Elizabeth R. Unger) to confirm the presence of malignancy. The first H&E slide was digitized using ScanScope XT (Aperio Technologies, Vista, CA, USA) at 0.25 hm per pixel resolution, equal to 40× objective. Of the 90 cases prepared, 5 lacking adequate tumor tissue and 2 with only *in situ* components were excluded.

Tissue from the remaining 83 cases was genotyped. One suitable tissue sample from each case was processed as previously described using high temperature assisted tissue lysis (19) and automated DNA purification with a Chemagic MSM1 (PerkinElmer, Waltham, MA, USA). The resulting 100 μl DNA elute was tested immediately or stored at −20°C until testing. A blank sample without tissue was included in every sample batch to monitor potential cross contamination. All DNA extracts were tested with the Linear Array HPV Genotyping Test (LA, Roche Diagnostics, Indianapolis, IN, USA), which distinguishes 37 different HPV genotypes (6, 11, 16, 18, 26, 31, 33, 35, 39, 40, 42, 45, 51, 52 (XR), 53, 54, 55, 56, 58, 59, 61, 62, 64, 66, 67, 68, 69, 70, 71, 72, 73, 81, 82, 83, 84, 89, IS39). Templates for the PCR reaction were prepared with 10 μl DNA and 40 μl H_2_O, otherwise following the manufacturer’s protocol. The reverse line-blot hybridization was performed with an automated platform; Beeblot instruments (Bee Robotics, Caernarfon, UK). Samples with negative or inadequate LA results were re-tested with the INNO-LiPA HPV Genotyping Assay (LiPA, Innogenetics, Gent, Belgium), which detects 29 HPV types including three types not covered by the Linear Array (43, 44, 74,) and performs a generic probe to identify other HPVs not on the array (HPV X). The assay was performed according to the manufacturer’s specifications using an Autoblot 3000 (MedTec, Buffalo Grove, IL, USA) for the line-blot procedure. Both typing assays included an endogenous positive control for the presence of amplifiable DNA. Samples negative for HPV and for the control probe in both assays were considered inadequate. Of the 83 penile cancer cases genotyped, 4 yielded inadequate results and were excluded.

A total of 79 cases were retained in the statistical analyses. Deidentified demographic (age, sex, population size), clinical (year of diagnosis, history of other cancers), pathologic (subsite, stage, grade), and outcome data (vital status, cause of death, survival time) were available from each registry. Stage was based on the SEER staging classification system (20). A large number of cases were histologically classified as unspecified squamous cell carcinoma (SCC). To improve the reliability of histological classification, digital images of all cases were reviewed and re-classified by one pathologist (Amy Powers) who was blinded to clinical and pathologic information as well as HPV status of cases.

Statistical analyses were conducted using SAS version 9.2. All tests were two-sided and a *p*-value of 0.05 was considered to be statistically significant. Overall HPV prevalence was based on the detection of one or more HPV genotypes in tumor tissue. Multiple genotypes detected in a case were not counted more than once in prevalence estimates. HPV 16, 18, 31, 33, 35, 39, 45, 51, 52, 56, 58, 59, 66, and 68 were considered high-risk genotypes (21). All other genotypes were considered to be of low or undetermined risk. Age was imputed to the mid-point of 5 year age groups for three cases from whom single year age was not available. Race/ethnicity was classified as non-Hispanic white, non-Hispanic black, Hispanic, and other. Comparisons by HPV status were made using the Chi-square test for discrete variables. Overall survival of HPV-positive and HPV-negative patients was compared using Kaplan–Meier and multivariable Cox regression analyses. Survival analyses excluded five cases for which vital status and survival time were not available.

## Results

Human papillomavirus DNA was detected in 50 of 79 (63%) invasive penile cancer cases. A total of 16 genotypes were detected (Table 1). HPV 16 was the most prevalent genotype comprising 72% (36/50) of HPV-positive cases, or 36 of 79 (46%) of all cases. Other high-risk types detected were HPV18, 31, 33, 35, 45, 52, 58, and 59. Two tumors were solely positive for low-risk types, HPV 6 and HPV 42, respectively. In total, genotypes other than HPV 16 or 18 were prevalent in 12 of 79 (15%) cases, or 24% of all HPV-positive tumors. One case was not positive for the 40 genotypes assayed and was designated HPV X. Multiple genotypes were found in 18% (9 of 50) of HPV-positive cases; HPV 16 was detected in 7 of these 9 cases.

**Table 1:** **Genotype distribution of HPV DNA-positive invasive penile cancer cases (*n* = 50.)**.

HPV types	Number of cases
6	1
16^a^	29
18^a^	2
33^a^	3
35^a^	1
42	1
45^a^	2
52^a^	1
6, 16^a^	1
16^a^, 45^a^	1
16^a^, 55	1
16^a^, 58^a^	1
16^a^, 18^a^, 72	1
16^a^, 18^a^, 31^a^, 58^a^, 73	1
16^a^, 45^a^, 55	1
33^a^, 62	1
35^a^, 53, 59^a^	1
X^b^	1

*^a^High-risk types; all others are considered to be of low or undetermined risk*.

*^b^HPV-positive for none of the 40 types assayed*.

Demographic and clinical characteristics of the 79 invasive penile cancer cases study cases in the study sample were comparable to that of all registry cases diagnosed over the same time period (data not shown). Study cases spanned a wide age range; 33–100 years (mean 70.9 ± 14.8). Whites comprised 56% of cases followed by blacks (24%), and Hispanics (17%). The large majority of cases were diagnosed in 2004–2005 (86%). Most cases were localized, early stage tumors (67%), and of moderately differentiated grade (51%). Thirty-two percent of tumors were of the glans or prepuce. A prior history of cancer was reported in 43% of cases; information on type of cancer was not available. Based on registry data, 61 of the 79 (76%) cases were histologically classified as SCCs not otherwise specified (SCC NOS), 17 were keratinizing SCC and 1 verrucous SCC. NOS cases consisted of those without histological subtype documented in the pathologic record. Following independent pathologic review, cases were re-classified as keratinizing SCC (*n* = 53), basaloid SCC (*n* = 3), warty SCC (*n* = 3), mixed basaloid-keratinizing SCC (*n* = 4), mixed warty-keratinizing SCC (*n* = 1), and verrucous SCC (*n* = 1); 13 remained SCC NOS (*n* = 13).

The detection of HPV (any genotype) in penile cancers did not significantly vary by age, race/ethnicity, population size of geographic region, stage, histology, grade, penile subsite, or cancer history (Table 2). Of penile cancers diagnosed during the most recent time period (2004–2005), 68% were HPV-positive compared with 36% of cases diagnosed in 1998–2003 (*p* = 0.05). In order to evaluate the possible influence of the age of specimens on the detection of HPV, we compared the number of samples with negative or inadequate results generated from the linear array test requiring retesting with the second assay by period of diagnosis. Four of 11 cases diagnosed in 1998–2003 were derived by LiPA compared to 10 of 67 cases diagnosed in 2004–2005 (*p* = 0.09).

**Table 2:** **Characteristics of invasive penile cancer cases by HPV status (*n* = 79)**.

Characteristics^a^	HPV−(*n* = 29)		HPV+ (*n* = 50)		*P*^b^
	No.	%^c^	No.	%^c^	
**AGE AT DIAGNOSIS (YEARS)**					
Mean (SD)	72.7 (SD 15.3)		69.8 (SD 14.5)		0.40
<60	4	21.1	15	78.9	0.27
60–79	14	41.2	20	58.8	
≥80	11	42.3	15	57.7	
**RACE/ETHNICITY^d^**					
White	15	37.5	25	62.5	0.77
Black	5	29.4	12	70.6	
Hispanic	5	41.7	7	58.3	
**POPULATION SIZE OF RESIDENCE**					
<20,000	16	32.0	34	68.0	0.42
20,000–250,0000	3	33.3	6	66.7	
250,000 ≥ 1,000,000	8	50.0	8	50.0	
**YEAR OF DIAGNOSIS**					
1998–2003	7	63.6	4	36.4	0.05
2004–2005	22	32.4	46	67.6	
**STAGE^e^**					
Localized	15	31.9	32	68.1	0.10
Regional or distant metastasis	12	52.2	11	47.8	
**HISTOLOGY^f^**					
Keratinizing SCC	23	43.4	30	56.6	0.19
Basaloid or warty SCC^g^	2	18.2	9	81.8	
Other SCC	4	26.7	11	73.3	
**TUMOR GRADE**					
Well-differentiated	10	43.5	13	56.5	0.46
Moderately differentiated	12	34.3	23	65.7	
Poorly differentiated	6	54.5	5	45.5	
**SUBSITE**					
Glans/prepuce	10	40.0	15	60.0	0.68
Other or unspecified	19	35.2	35	64.8	
**HISTORY Of OTHER CANCERS**					
No	16	35.6	29	64.4	0.81
Yes	13	38.2	21	61.8	

*^a^Missing data: race/ethnicity (*n* = 7); population size of residence (*n* = 4); stage (*n* = 9); grade (*n* = 10)*.

*^b^Chi-square test comparing HPV− and HPV+*.

*^c^% Of total for each variable category (row)*.

*^d^Whites and blacks include non-Hispanics only; Asians excluded due to frequencies <5*.

*^e^Stage classified according to SEER Summary Stage 2000*.

*^f^Based on independent review by one pathologist*.

*^g^Includes tumors with basaloid or warty types combined with other histologies*.

**Figure 1:**
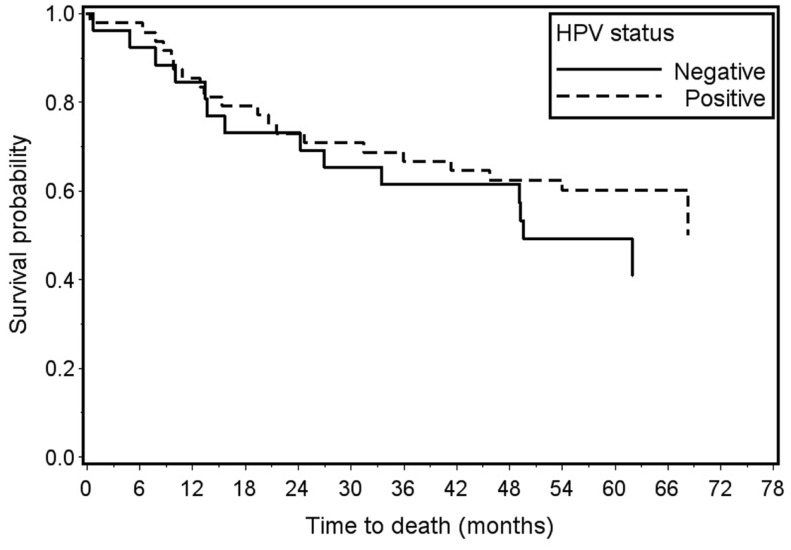
**Overall survival of invasive penile cancer cases by HPV status: any HPV+ (*n* = 48) vs. HPV− (*n* = 26) Log-rank *p*-value = 0.37**.

Survival analyses included 74 of the 79 penile cancer cases; 5 cases without available outcome data were excluded. Overall survival was 60% for HPV-positive cases and 50% for HPV-negative cases (log-rank *p*-value = 0.37) (Figure 1). Survival did not vary significantly by HPV status when limited to high-risk types or HPV 16 alone (data not shown). Proportional hazards assumptions were met. For any HPV, the unadjusted hazard ratio was 0.73, 95% CI 0.37–1.45. After adjustment for age, stage, and year of diagnosis, the hazard ratio was 0.86, 95% CI 0.40–1.86. In both univariate and multivariate models, high-risk HPV and HPV 16 status did not predict survival (data not shown).

## Discussion

This is the first study of HPV prevalence in invasive penile cancer cases drawn from diverse regions of the U.S., including those with large Hispanic, black, and Asian populations. Although not a representative sample of the entire U.S. population, cases came from seven cancer registries covering defined geographic regions of the country, including five registries with statewide coverage. The overall HPV prevalence of 63% in this U.S. sample is higher than estimates of HPV prevalence in penile cancers worldwide (2, 3, 5). Two reviews of published studies of penile cancers diagnosed worldwide in 1986–2008 found an overall HPV prevalence of 47–48% (2, 5). Differences in the sensitivity of laboratory methods may account for a higher prevalence in our study population. Notably, the present study utilized methods designed to enhance DNA isolation from fixed, archival tissue, and two separate PCR-based genotyping assays detecting a broad range of HPV genotypes. HPV prevalence may have also been influenced by unmeasured factors specific to the study population such as sexual risk factors and circumcision status.

Our findings agree with previous studies demonstrating HPV 16 to be the predominant genotype in penile cancers (2, 6, 7). HPV 16 and 18, the two high-risk types covered by current prophylactic vaccines, were identified in nearly half of the tumors. The potential role of other genotypes in penile carcinogenesis is underscored by the detection of 14 other types, including those considered to be low-risk. The detection of low-risk types HPV 6 and 42 alone in a small proportion of penile tumors is consistent with previous studies and may be indicative of the rare carcinogenic potential of these types (5, 22–24).

Regional and racial/ethnic disparities in the incidence of penile cancer have been observed in the U.S. with higher incidence in the South and among blacks and Hispanics and lower incidence in the West and among Asian-Pacific Islanders (1, 25, 26). In our limited sample, we observed no differences between HPV-positive and HPV-negative penile cancer cases by age, race/ethnicity, or population size of geographic region.

We also observed no significant differences in HPV status by stage, histology, grade, or penile subsite. Notably, nearly all basaloid and warty tumors, including mixed subtypes, were HPV-positive. This is consistent with previous studies demonstrating a higher prevalence of HPV in these histological types (2, 4, 7).

The incidence of invasive penile cancer in the U.S. has significantly declined over the past several decades (25, 26). The reason for this decline is unknown but may be attributed to population-level changes including decreases in smoking rates (27) and increases in circumcision in older birth cohorts (28). A major limitation of this study is the lack of patient information on other risk factors for penile cancer including sexual history, smoking, circumcision, and phimosis (4, 8–11). This information is generally not abstracted by cancer registries. The availability of such data would permit a more comprehensive evaluation of the role of HPV relative to these factors.

Human papillomavirus was detected more frequently in the recently diagnosed cases. A recent study reported that the prevalence of HPV in U.S. oropharyngeal cancers significantly increased over calendar time from 1984 to 2004 (29). It was surmised that this may reflect increases in high-risk sexual activity and HPV exposure over time. The authors corrected for potential loss in assay sensitivity with specimen age. In the present study, prevalence estimates were not corrected. Therefore, it is possible that the observed temporal differences in HPV prevalence were an artifact due to the relative age of the archival tissue specimens. That is, viral DNA may have been less readily detected in older specimens due to degradation of nucleic acid in preserved tissue over time. While we found no evidence for this, it is hard to completely exclude this explanation. The ability to examine temporal trends in the present study is limited by the relatively narrow (8 year) period of diagnosis of cases. In addition, because registries used different temporal sampling frames, it is possible that observed temporal differences reflect geographic differences. In the present study, HPV prevalence is not presented by registry to protect patient confidentiality given the small number of cases.

In patients with oropharyngeal cancer HPV tumor positivity predicts a favorable outcome, including overall survival and disease-free survival (30–36). Few studies have examined the role of HPV in penile cancer survival. Lont et al. (6) observed that penile cancer patients with high-risk human HPV had significantly higher 5-year survival, and HPV was an independent predictor of survival after adjustment for age and clinical characteristics (6). We found no association of HPV status, including any HPV and HPV 16, with penile cancer survival.

As with other PCR-based studies, we cannot be certain that the HPV DNA detected was present and causal in the tumor. HPV infection could have been present in nearby tissue. Evaluation of other molecular markers of HPV, including p16^INK4a^ and E6/E7 mRNA, may provide important insight into the clinical relevance of viral detection in penile cancers (37).

Forty-eight percent of penile cancers were positive for HPV 16 or 18, which are included in current vaccines. HPV vaccination has demonstrated high efficacy for the prevention of genital HPV infection and external genital lesions in males (38). Nonetheless, vaccine efficacy for the prevention of penile cancers has not yet been directly evaluated in clinical trials.

The relatively high prevalence of HPV in our study population provides limited evidence of a more prominent and, possibly, increasing role of infection in penile carcinogenesis in the U.S. compared to other parts of the world. Population differences in the prevalence of sexual risks, circumcision, and other factors influencing HPV acquisition and persistence may account for these results. Our findings would be bolstered by future U.S. studies which include larger, population-based samples, which are derived from patients diagnosed with penile cancer over a wider time period.

## Conflict of Interest Statement

Brenda Y. Hernandez has received consultation from Merck and Co., Inc., for activities unrelated to this research project. The other co-authors declare that the research was conducted in the absence of any commercial or financial relationships that could be construed as a potential conflict of interest.
